# Transcriptome analysis and genome-wide identification of the dehydration-responsive element binding gene family in jackfruit under cold stress

**DOI:** 10.1186/s12864-024-10732-1

**Published:** 2024-09-04

**Authors:** Xiangwei Ma, Pengjin Zhu, Yingjun Du, Qiqi Song, Weiyan Ye, Xiuguan Tang, Jiang He, Yunjie Zhong, Jingli Ou, Xinhua Pang

**Affiliations:** 1 Present Address: Guangxi Subtropical Crops Research Institute , Nanning, 530000 China; 2 Guangxi Key Laboratory of Quality and Safety Control for Subtropical Fruits, Nanning, 530000 China

**Keywords:** Jackfruit, Dehydration responsive element binding (DREB), Expression profile, Cold stress

## Abstract

**Background:**

Jackfruit (*Artocarpus heterophyllus* Lam.) is the world’s largest and heaviest fruit and adapts to hot, humid tropical climates. Low-temperature injury in winter is a primary abiotic stress, which affects jackfruit growth and development. Therefore, breeding cold-resistant varieties and identifying the vital genes in the process of cold resistance are essential. The dehydration-responsive element binding (DREB) gene family is among the subfamily of the *APETALA2/ethylene response factor* transcription factor family and is significant in plant abiotic stress responses.

**Methods:**

In this study, a comparative analysis of the cold resistance property of ‘GuangXi’ (‘GX’) and ‘Thailand’ (‘THA’) jackfruit strains with different cold resistance characteristics was performed through chlorophyll fluorescence and transcriptome sequencing.

**Results:**

We found that differentially expressed genes (DEGs) are significantly enriched in the metabolic processes. Here, 93 *DREB* genes were identified in the jackfruit genome, and phylogenetic analysis was used to classify them into seven groups. Gene structure, conserved motifs, chromosomal location, and homologous relationships were used to analyze the structural characteristics of the *DREB* family. Transcriptomics indicated that most of the AhDREB genes exhibited down-regulated expression in ‘THA.’ The DEGs *AhDREB12*, *AhDREB21*, *AhDREB29*, and *AhDREB34* were selected for quantitative real-time PCR, and the results showed that these genes also had down-regulated expression in ‘THA.’

**Conclusions:**

The above results suggest the significance of the *DREB* family in improving the cold resistance property of ‘GX.’

**Supplementary Information:**

The online version contains supplementary material available at 10.1186/s12864-024-10732-1.

## Background

The frequent occurrence of cold damage in winter is a significant factor affecting plant growth and development, and the selection of plant varieties with strong cold resistance remains challenging in production [1, 2]. The expression of transcription factors (TFs) is crucial in these complex processes [3, 4]. TFs are *trans*-acting factors, which are specific proteins that can bind to *cis*-acting elements associated with gene promoter regions to activate or inhibit gene expression [5]. Dehydration-responsive element binding protein (DREB), which belongs to the APETAL2/ethylene responsive element binding factor subfamily APETALA2/ethylene response factor (*AP2/ERF*), has been identified as a major regulator of cold adaptation in many angiosperms [6–9]. The *D*REB family contains a conserved AP2 domain of 57–70 amino acid residues, regulates the expression of various downstream stress genes, and endows plants with stress tolerance by interacting with the dehydration-responsive element/C-repeat (DRE/CRT) in the promoter regions of stress-inducing genes [6]. An increasing number of studies have shown that the function of *DREB* in cold resistance is conserved in maize [10], *Brassica napus* [11], barley [12], and other higher plants [13]. The DREB is also known as the C-repeat binding factor (CBF) protein [14]. By integrating multi-omics data from genomes, transcriptomics, and CBFs/DREB1s whole-genome binding profiles, Nie et al. [15] proposed that CBFs/DREB1s may be an evolutionary strategy for angiosperms to adapt to global cooling. Song et al. [16] focused on the CBF-dependent pathway during cold domestication in *Arabidopsis*. CBFs, as an essential signal integrator, were found to be critical in the early response of *Arabidopsis* to cold stress by activating target gene transcription [16]. AtCBF1, AtCBF2, and AtCBF3 are distributed in tandem on Chromosome 4 of *Arabidopsis*, and CBF1 or CBF3 knockout causes the sensitivity of downstream target genes to freezing stress [17]. Chen et al. [18] isolated *DREB* from soybeans through rapid amplification of cDNA ends, and Northern blotting indicated that *DREB3* participates in an abscisic acid-independent cold stress-responsive signaling pathway. Furthermore, cold resistance was enhanced in *GmDREB3*-overexpression plants, and fresh weight and osmolality in these plants were higher than those in wild-type plants under cold stress. These results indicate the significance of the *DREB* family in plant responses to cold stress. Therefore, identifying the DREB gene family in plants and revealing their stress resistance functions and pathways are essential.

Jackfruit (*Artocarpus heterophyllus* Lam.) is a tropical climacteric fruit belonging to the Moraceae family and is a popular tropical fruit planted in the tropical and subtropical areas of China, with a planting area exceeding 500,000 acres [19, 20]. Jackfruit adapts to hot and humid tropical climates, and temperature is the crucial factor affecting its growth and development. When the temperature is below 5–7 ℃, it does not easily bloom and fruit [16]. The scale of jackfruit planting in Guangxi Province, China, has been continuously expanding recently; however, cold damage has become a significant factor limiting the development of the jackfruit industry in Guangxi [21]. Mining germplasm resources with strong cold to provide parent or donor genes has become a focus in stress-resistant jackfruit breeding. Therefore, identifying crucial pathways and genes in jackfruit with strong cold resistance will enable high-efficiency screening of jackfruit germplasm resources for cold stress resistance. In a previous study, cold-tolerant (‘GX’) and cold-sensitive (‘THA’) varieties were selected through performance observation and physiological index determination [16]. ‘Guangxi’ (‘GX’) is a local variety in Guangxi Province (China) that has good cold resistance and can grow normally even under a low temperature of 3 ℃. ‘Thailand’ (‘THA’) is a variety introduced from Thailand with good fruit quality and is currently a mainstream variety in the market. However, the cold sensitivity of ‘THA’ limits its cultivation and promotion. Therefore, ‘GX’ and ‘THA’ are good materials for studying the cold resistance property of jackfruit. The *DREB* family is also significant during jackfruit response to cold stress, with the ‘S10’ cultivar genome facilitating thestudy of the effect of the *DREB* family on cold tolerance in the species. However, the *DREB* family in jackfruit remains uninvestigated.

We used transcriptomics to analyze the primary metabolic pathways and differentially expressed genes (DEGs) of two jackfruit cultivars, ‘Guangxi’ (‘GX’) and ‘Thailand’ (‘THA’), under cold stress. The *DREB* family from the DEGs was screened, and 93 jackfruit *DREB* genes were identified. Basic parameters, phylogenetic relationships, gene structure, motif composition, chromosomal distribution, gene duplication, and expression patterns under cold stress were analyzed. The results expand the understanding of *DREB* genes in jackfruit and have significance for the improvement and breeding of cold-resistant jackfruit varieties.

## Methods

### Plant materials

To explore the adaptability of jackfruit in response to cold stress, two jackfruit cultivars, ‘GX’ (GXRZBLM00051) and ‘THA’ (GXRZBLM00013), preserved in the jackfruit germplasm resource nursery of the Guangxi Subtropical Crops Research Institute (22.899°N, 108.343°E), were selected for field sampling and follow-up. After exposure to low-temperature stress under natural conditions (between February 19 and 24, 2022), there was continuous low temperature in Nanning, with daily minimum and maximum temperatures of 3 and 15 ℃, respectively. For jackfruit tree crown temperature, the daily minimum and maximum temperatures were 3.5 and 14 ℃, respectively. The leaves of the two cultivars were collected (the first leaf on each branch was counted from top to bottom). We achieved all required permits for the protected areas from the local governments and relevant institution. This research was carried out in compliance with the relevant laws of Nanning, Guangxi, China. The materials collected were divided into two parts. One part was used for chlorophyll fluorescence imaging, and the other was stored at -80 ℃ for transcriptomic sequencing and RNA extraction. The temperatures were measured using an environmental Meteorological Automatic Monitoring system (RR-9150, Beijing, Yugen Technology) and a temperature recorder (COS-04, Shandong Jianda Renke Electronic Technology).

### Identification and classification of the AhDREB gene family

The genome of the jackfruit cultivar ‘S10’ [21] was downloaded from the National Center for Biotechnology Information Sequence Read Archive database (PRJNA788174 and PRJNA791757). Sequence information for the *AP2/ERF* family of *Arabidopsis* was downloaded from the online TAIR database (https://www.arabidopsis.org/index.jsp) and used as the probe sequence (e-value < 0.001) for the Basic Local Alignment Search Tool (BLAST). The sequence of the AP2/ERF conserved domain (PF00847) was downloaded from the Protein Family Analysis and Modeling (Pfam) website (http://pfam.xfam.org/) and used to further identify *AP2/ERF* family members (e-value < e − 5). The conserved domain of AP2/ERF was confirmed using the online Simple Modular Architecture Research Tool [22] (http://smart.embl.de/), and biosequence analysis was performed using profile hidden Markov models (https://www.ebi.ac.uk/Tools/hmmer/) [23]. Strawberry (*Favaria vesca*) DERB sequences were downloaded from the Genome Database for Rosaceae [24]. Phylogenetic trees of AP2/ERF in jackfruit and DREB in strawberry and jackfruit were constructed using the neighbor-joining method and 1,000 bootstrap replicates in MEGA [25], respectively. The AP2/ERF and AP2/EREBP proteins of jackfruit and *Arabidopsis*, respectively, were compared and classified into different groups (RAV, AP2, ERF, and DREB). The fourteenth valine, the nineteenth glutamic acid, and AP2 domains were the standards for distinguishing the DREB gene family. The amino acids, theoretical isoelectric point, and molecular weight were analyzed using Expasy ProtParam (https://web.expasy.org/protparam/), and subcellular localization was predicted using WoLF PSORT (https://www.genscript.com/wolf-psort.html).

### Gene structure and conserved motif analysis of the AhDREB gene family

The full-length sequences of the *DREB* family and jackfruit genome files were used to build gene structures using the Gene Structure Display Server 2.0 [26]. Conserved motif analysis of the *DREB* family was conducted with Multiple Em for Motif Elicitation (MEME) [27] (https://meme-suite.org/meme/index.html) using the default parameters, and the maximum number of pattern parameters was set to 20.

### Chromosomal localization and synteny analyses

The chromosomal localization information of the *DREB* family was retrieved from the jackfruit genome files and graphically represented using Tbtools v2.031 [28]. Gene Location Visualization from GTF/GFF program. The syntenic gene pairs of AhDREB genes were analyzed using One Step MCScanX; BLASTP results and gene location information were used for the next step input of Tbtools [29]. The proximal, dispersed tandem of AhREB family genes was identified using Tbtools, and the results were visualized using Tbtools Advanced Circos [30].

### Chlorophyll fluorescence assay

‘GX’ and ‘THA’ leaves were used to analyze chlorophyll fluorescence parameters. The leaves were darkened for 30 min and placed in a chlorophyll fluorescence (CF) imager (Technologica, Colchester, UK) to determine the initial fluorescence (Fo) and maximum fluorescence (Fm). After 30 min of full-light adaptation, the Fo’, Fm’, and steady-state fluorescence (F’) were measured, and fluorescence parameters, such as maximum quantum yield of Photosystem II (Fv’/Fm’), non-photochemical quenching (NPQ), and the effective quantum yield of Photosystem II (Fq’/Fm’) were controlled and calculated using the software.

### RNA sequencing, data, and quantitative real-time PCR (qRT-PCR) analysis

Total RNA was extracted from ‘GX’ and ‘THA’ leaves (each sample was prepared in triplicate), and RNA integrity and purity were assessed through agarose gel electrophoresis and spectrophotometry (NanoPhotometer; Implen, Munich, Germany). RNA concentration was measured using the Qubit 2.0 fluorometer (Thermo Scientific, USA), and RNA integrity was measured with Agilent 2100 bioanalyzer (HACH, USA). The enriched mRNA was broken into short fragments; the first cDNA strand was synthesized using the short fragment mRNA as the template, and the second cDNA strand was synthesized using the first cDNA strand as the mold plate by adding buffer solution, dNTPs, and DNA polymerase. After purification, end repair, the addition of poly (A) tail, and connection of sequencing joints, AMPureXPbeads were used for fragment size selection, and PCR enrichment was performed to obtain the final cDNA library. After qualification, the library was sequenced using an Illumi-TMnaHiSeq platform. Trinity software was used to concatenate clean reads, and Corset hierarchical clustering was used to obtain the longest Cluster sequence, which was further analyzed as Unigene. DIAMOND BLASTX software was used to compare Unigene with Kyoto Encyclopedia of Genes and Genomes (KEGG), RefSeq non-redundant proteins (NR), Swiss-Prot, Gene Ontology (GO), clusters of orthologous groups (COG) / euKaryotic Ortholog Groups (KOG), and TrEMBL databases to predict the amino acid sequence of Unigene. Subsequently, the HMMER software was compared with the Pfam database, and finally the annotation information of Unigene was obtained. The transcriptome sequencing analysis of six samples was completed, and 41.12 Gb of Clean Data was obtained. The Clcan Data of each sample reached 6 GB, and the percentage of Q30 bases was *≥* 92%.

The RNA sequencing of ‘GX’ and ‘THA’ was performed using the HiScript^®^ Q RT SuperMix for qPCR (+ gDNA wiper) (Vazyme, Nanjing, China) to synthesize cDNA, which was used as a template for amplification after 10-fold dilution. The expression levels of *DREB* genes associated with cold stress response were determined through qRT − PCR on a QuantStudio™ 5 Real-Time PCR System (Applied Biosystems, Foster City, CA, USA). Reaction system 20 μL: upstream and downstream primers (10 μmol/L) 1μL, dd H2O 7 μL, 1 μL cDNA, and SYBR Premix Ex Taq 10 μL. Reaction procedure: predenaturation at 95 ℃ for 30 s; denaturation at 95 ℃ for 10 s, annealing at 60 ℃ for 30 s, extension at 72 ℃ for 30 s, a total of 50 cycles, and three sets of repeats were performed. The results were calculated using the 2^−^∆∆Ct method.

### Statistical analysis

Significant differences were determined using a one-way analysis of variance with Duncan’s test. The data were imported into IBM SPSS Statistics 16.0 to analyze significant differences (Student’s t-test, *p* < 0.01, ***p < 0.001, and ****p < 0.0001) and were graphically displayed using GraphPad Prism 9. The differential expression analysis of transcriptome was performed using the DESeq2 package with the criteria of fold change |1og2Fold Chang| ≥ 1 and FDR (False Discovery Rate) < 0.01.

## Results

### Phenotypic differences in ‘GX’ and ‘THA’ under cold stress

**Fig. 1:**
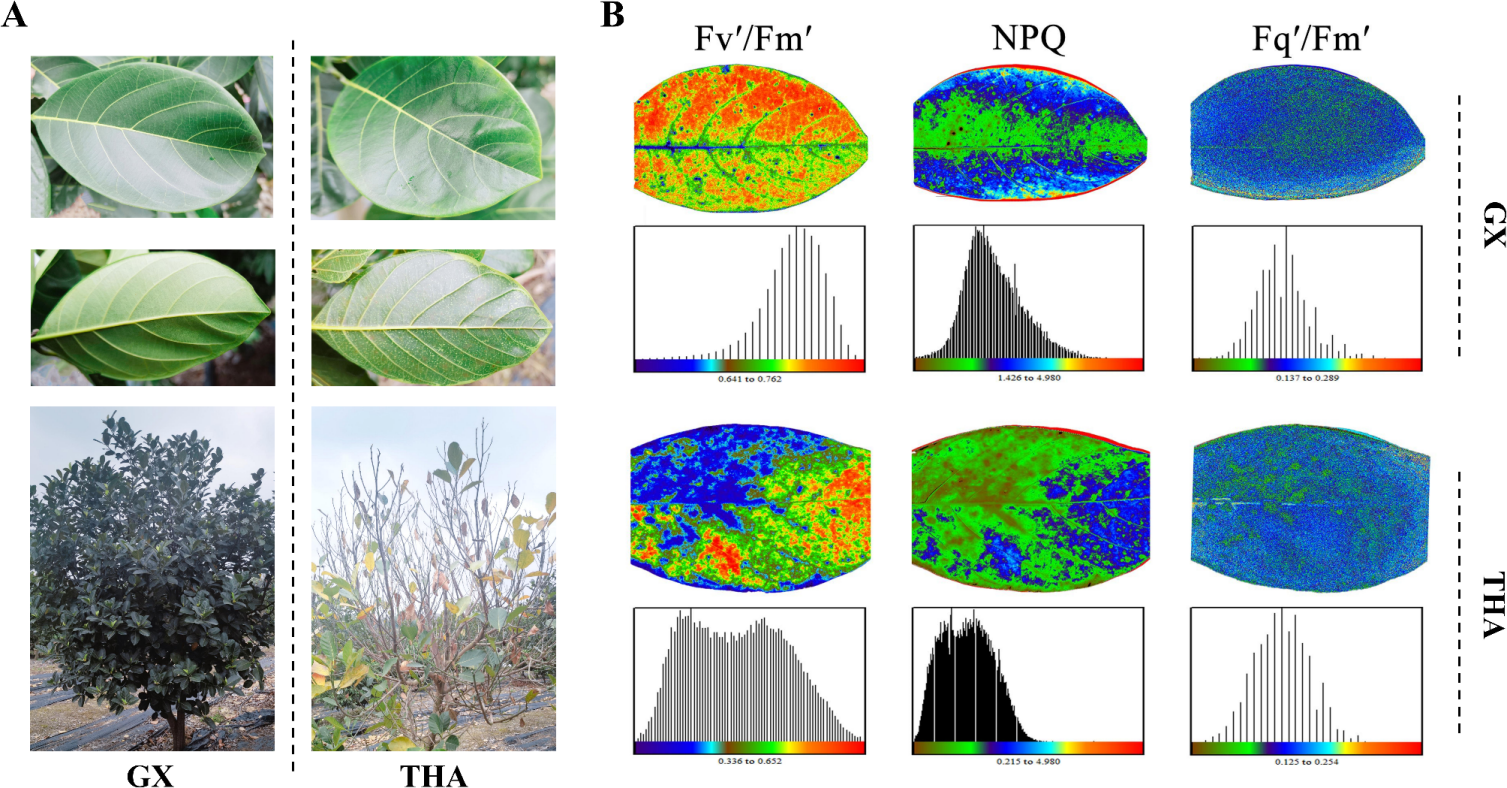
Evaluation of cold resistance in different jackfruit phenotypes ‘Guangxi’ (‘GX’) and ‘Thailand’ (‘THA’). (**A**) Phenotypic characteristics of jackfruit under cold stress. Left: jackfruit of ‘GX’ variety. Right: jackfruit of ‘THA’ (**B**) Chlorophyll fluorescence assay of jackfruit after cold stress. Fv’/Fm’: photochemicalefficiency of photosystemII in the light. Fqʹ/Fmʹ: NPQ: non-photochemical quenching. The effective quantum yield of PSII

A phenotypic difference between ‘GX’ and ‘THA’ plants was observed under cold stress (Fig. 1A). The leaves of the ‘GX’ jackfruit were dark green with no apparent damage to their surface and back, whereas those of the ‘THA’ jackfruit were green with small dotted brown spots on their back. The leaves of the ‘GX’ trees were thicker than those of the ‘THA’ trees, indicating that the resistance to cold stress in the ‘GX’ trees was stronger than that in the ‘THA’ trees. To further verify this result, CF imaging was used to investigate the response of photosynthetic energy dissipation to cold stress in ‘GX’ and ‘THA.’ Chlorophyll fluorescence parameters Fvʹ/Fmʹ, Fqʹ/Fmʹ, and NPQ were considered significant indices to evaluate plant stress resistance during early stage [31]. The CF parameters showed significant differences between ‘GX’ and ‘THA’ (Fig. 1B); the fluorescent signals Fvʹ/Fmʹ and Fqʹ/Fmʹ in ‘GX’ were stronger than that in ‘THA,’ and the same was true of the corresponding values, indicating that cold stress caused a decreased proportion of excitation energy used for photochemical pathways in ‘THA.’ The fluorescent signal of NPQ in ‘THA’ was weaker than that in ‘GX,’ indicating that the ability of ‘THA’ to dissipate superheated excitation energy was weaker than that of ‘GX.’ These results show that ‘GX’ is more resistant to cold than ‘THA.’

### ‘GX’ regulates resistance to cold stress through metabolic pathways

**Fig. 2:**
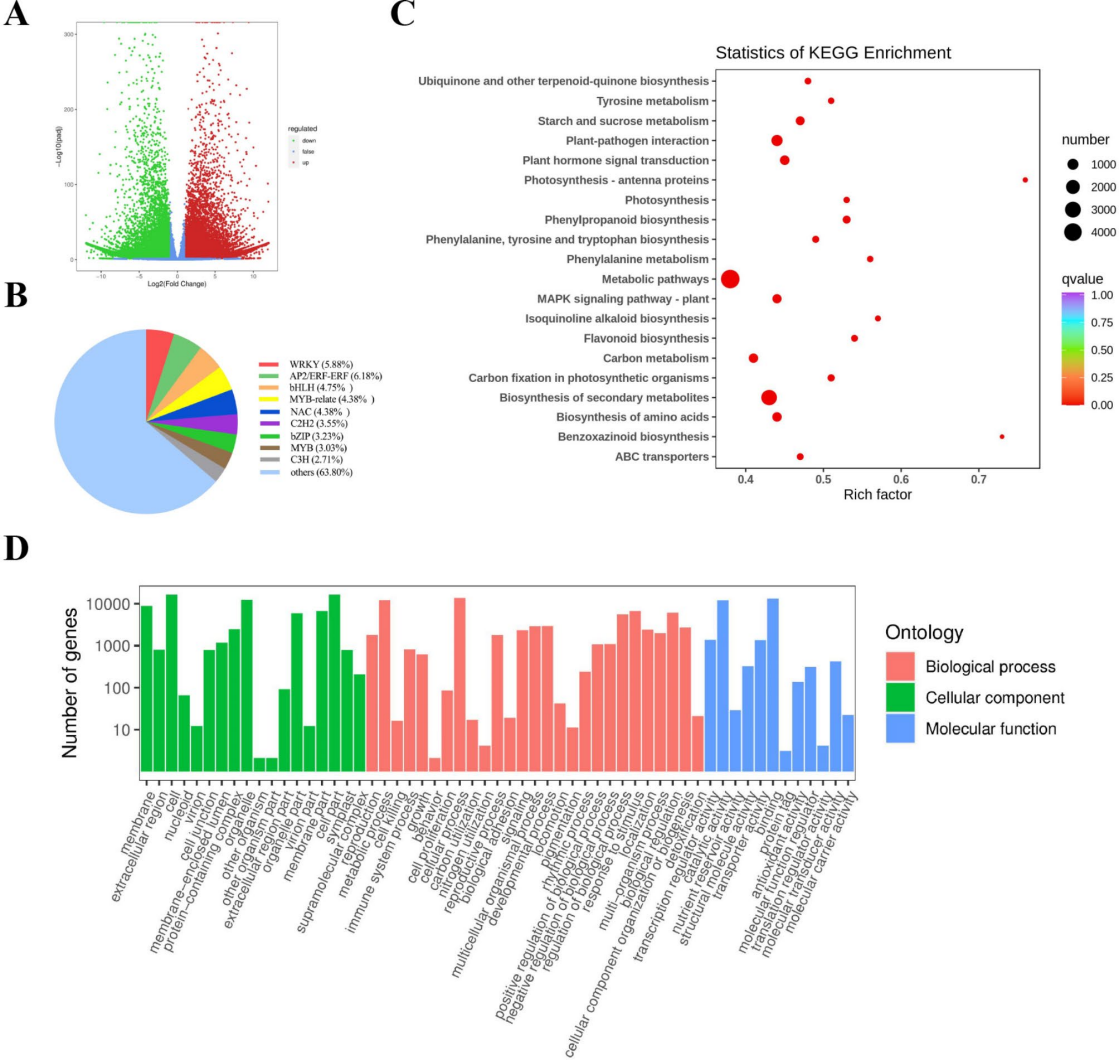
Transcriptome data analysis of different jackfruits under cold stress. (**A**) Number of up- or down-regulated differentially expressed genes (DEGs) in the comparison between ‘GX’ and ‘THA’ jackfruits. (**B**) The pie chart of transcription factors in different families. (**C**) The Kyoto Encyclopedia of Genes and Genomes (KEGG) enrichment analysis of DEGs in ‘GX’ vs. ‘THA’. (**D**) Gene ontology (GO) enrichment analysis. |1og2Fold Chang| ≥ 1, FDR < 0.01

To further investigate the mechanism of ‘GX’ resistance to cold stress, RNA-seq was performed on ‘GX’ and ‘THA’ leaves. Pearson’s correlation coefficient showed good reproducibility among the three biological replicates (Figure S1). The FDR < 0.05 and ¦log2 Fold Change¦ ≥1 were used to identify the DEGs. Among these DEGs, 17,083 and 13,502 were up- and down-regulated, respectively (Fig. 2A). A study [32] has shown that plants can improve their resistance to cold stress by regulating certain metabolic pathways and metabolism-related complexes to get a steady-state balance. KEGG and GO analyses revealed that the DEGs were significantly enriched in metabolic processes (Fig. 2C, D), including “tyrosine metabolism,” “starch and sucrose metabolisms,” “phenylpropanoid biosynthesis,” “phenylalanine metabolism,” “metabolic pathways,” “carbon metabolism,” and “biosynthesis of secondary metabolites.” Thus, we speculated that DEGs might be involved in certain metabolic pathways that increase resistance to cold stress in ‘GX.’ Moreover, there were differentially expressed 1,617 TFs, and the AP2/ERF genes were the most abundant (Fig. 2B), suggesting the significance of AP2/ERF genes in ‘GX’ resistance to cold stress.

### Identification of AP2/ERF family genes in jackfruit

**Fig. 3:**
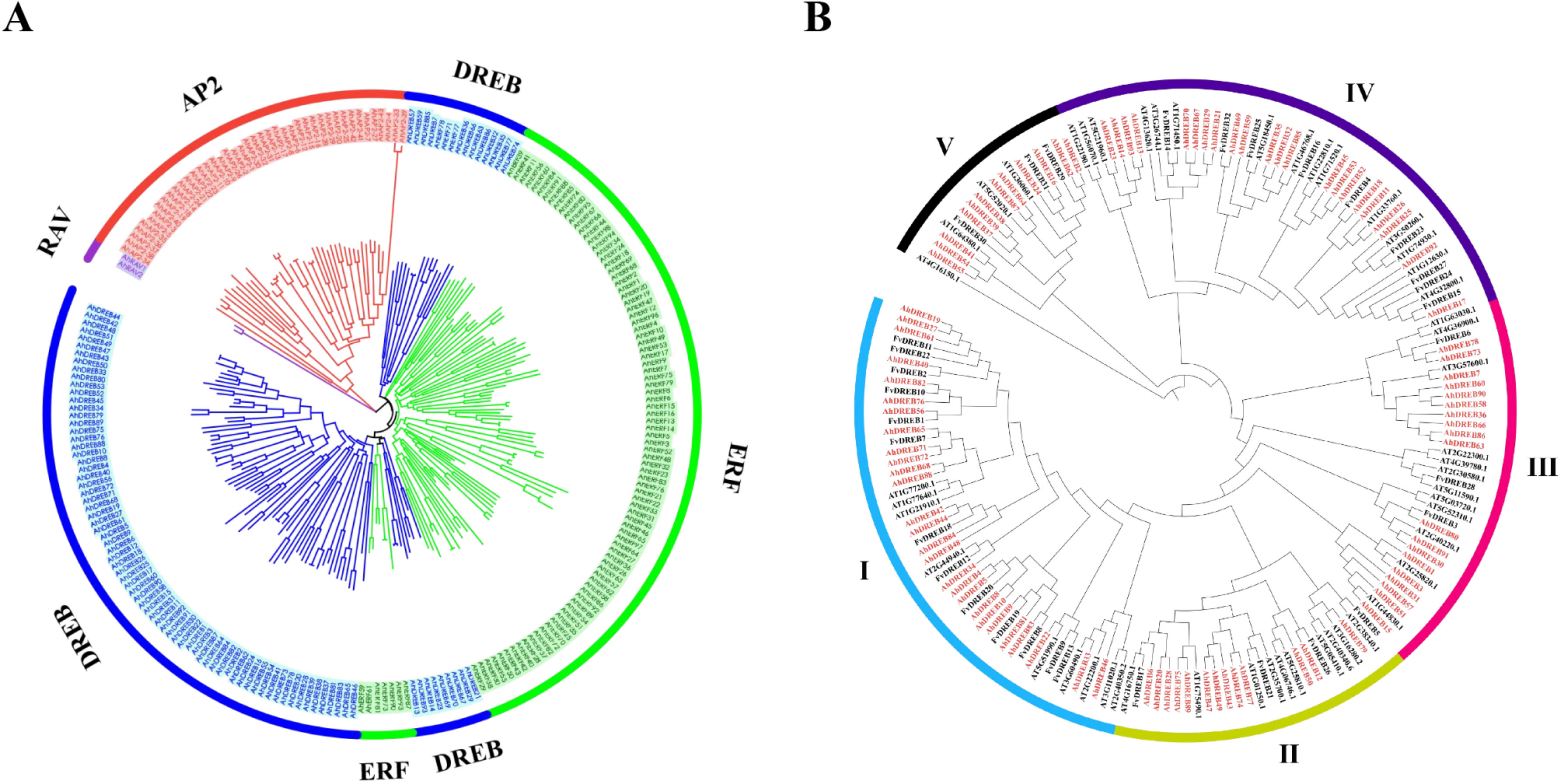
Phylogenetic tree analysis of the APETALA2/ethylene response factor (AP2/ERF) family and dehydration-responsive element binding protein (DREB) subfamily proteins. (**A**) The phylogenetic tree of AP2/ERF proteins from jackfruit. Different colors represent different subgroups. (**B**) Phylogenetic tree of DREB subfamily proteins from jackfruit, Arabidopsis, and strawberry. The color of the gene ID of jackfruit was red; I, II, III, IV, and V represent different subfamilies

Based on the number of conserved AP2 domains and motif similarity, the *AP2/ERF* family of TFs was divided into DREB, ERF, AP2 (APETALA2), RAV (related to ABI3/VP), and unclassified factors [33]. The phylogenetic tree was used to classify the jackfruit AP2/ERF proteins into four typical subfamilies (Fig. 3A), namely AP2, RAV, ERF, and DREB, which comprised 2, 43, 95, and 93 proteins, respectively. The jackfruit genome was scanned, and 93 *DREB* genes were identified using BLAST with Arabidopsis *DREB* genes. The annotations of the 93 identified *DREB* genes were named according to their chromosomal positions. Basic parameters were analyzed to explore the functions of the *DREB* family (Table 1). The number of amino acids in the DREB family protein sequences ranged from 132 (AhDREB58) to 432 (AhDREB64), the theoretical isoelectric points ranged from 4.57 (AhDREB11) to 10.87 (AhDREB50), and the molecular weights ranged from 14.91 KDa to 48.63 KDa. Subcellular localization analysis indicated that most AhDREB family proteins were localized in the nucleus, whereas other members were localized in the cytoplasm and nucleus.

**Table 1:** Basic parameter analysis of the AhDREB gene family

Gene Name	Gene ID	Chromosome location	Group	No. amino acids	pI	MW	Subcellular localization
*AhDREB1*	AHE.Chr01.887	Chr01:16732611–16,733,174	A-5	187	9.48	20714.92	Cytoplasm. Nucleus.
*AhDREB2*	AHE.Chr02.303	Chr02:10543402–10,545,288	A-6	335	5.61	37062.81	Nucleus.
*AhDREB3*	AHE.Chr02.699	Chr02:19782345–19,783,914	A-5	175	7.72	18808.83	Cytoplasm.
*AhDREB4*	AHE.Chr03.167	Chr03:1762897–1,764,301	A-4	277	5.42	30235.92	Cytoplasm. Nucleus.
*AhDREB5*	AHE.Chr03.226	Chr03:2397189–2,398,423	A-4	305	5.34	32624.83	Cytoplasm. Nucleus.
*AhDREB6*	AHE.Chr03.745	Chr03:8177686–8,178,705	A-4	213	5.06	22951.52	Cytoplasm. Nucleus.
*AhDREB7*	AHE.Chr04.1522	Chr04:24027287–24,028,144	A-6	285	8.67	31293.69	Nucleus.
*AhDREB8*	AHE.Chr04.188	Chr04:2581076–2,582,421	A-4	271	5.42	29699.28	Nucleus.
*AhDREB9*	AHE.Chr04.352	Chr04:4586969–4,588,725	A-4	282	4.99	30447.3	Nucleus.
*AhDREB10*	AHE.Chr04.47	Chr04:620029–621,381	A-4	273	5.31	29908.56	Nucleus.
*AhDREB11*	AHE.Chr04.618	Chr04:7160220–7,161,285	A-5	206	4.57	22844.11	Cytoplasm. Nucleus.
*AhDREB12*	AHE.Chr04.896	Chr04:11516658–11,517,667	A-4	206	5.06	22276.83	Nucleus.
*AhDREB13*	AHE.Chr05.1186	Chr05:24946573–24,947,453	A-6	198	9.37	21751.35	Nucleus.
*AhDREB14*	AHE.Chr06.735	Chr06:18582228–18,583,457	A-6	192	9.2	21195.87	Nucleus.
*AhDREB15*	AHE.Chr07.1004	Chr07:10742057–10,742,971	A-5	277	4.94	31273.74	Cytoplasm. Nucleus.
*AhDREB16*	AHE.Chr07.1284	Chr07:13780770–13,783,523	A-6	361	6.4	39663.36	Nucleus.
*AhDREB17*	AHE.Chr07.1499	Chr07:16470647–16,471,240	A-4	197	5.29	21520.07	Nucleus.
*AhDREB18*	AHE.Chr07.1503	Chr07:16509996–16,510,826	A-4	213	5.64	23778.61	Cytoplasm. Nucleus.
*AhDREB19*	AHE.Chr07.2425	Chr07:36171251–36,171,979	A-4	242	5.88	26039.94	Nucleus.
*AhDREB20*	AHE.Chr07.342	Chr07:4010841–4,011,617	A-2	258	6.05	28158.15	Nucleus.
*AhDREB21*	AHE.Chr07.586	Chr07:6272941–6,275,552	A-6	206	9.03	23230.25	Nucleus.
*AhDREB22*	AHE.Chr07.756	Chr07:8150352–8,152,462	A-5	181	7.86	20427.55	Cytoplasm. Nucleus.
*AhDREB23*	AHE.Chr08.1170	Chr08:12516982–12,518,454	A-6	271	7.2	30231.5	Nucleus.
*AhDREB24*	AHE.Chr08.1239	Chr08:13439343–13,441,558	A-6	363	7.14	39618.26	Nucleus.
*AhDREB25*	AHE.Chr08.1414	Chr08:15168275–15,168,979	A-4	197	5.26	21374.94	Nucleus.
*AhDREB26*	AHE.Chr08.1415	Chr08:15181865–15,182,509	A-4	214	5.45	23741.52	Nucleus.
*AhDREB27*	AHE.Chr08.2105	Chr08:30674013–30,675,096	A-4	244	6.23	26385.3	Nucleus.
*AhDREB28*	AHE.Chr08.251	Chr08:2465072–2,466,427	A-2	257	8.86	27640.58	Nucleus.
*AhDREB29*	AHE.Chr08.401	Chr08:3964741–3,967,960	A-6	140	10.4	15621.41	Nucleus.
*AhDREB30*	AHE.Chr08.690	Chr08:7013331–7,013,879	A-5	182	7.87	20612.87	Cytoplasm. Nucleus.
*AhDREB31*	AHE.Chr08.919	Chr08:9626628–9,627,443	A-5	271	4.77	30549.02	Cytoplasm. Nucleus.
*AhDREB32*	AHE.Chr09.2000	Chr09:30020435–30,021,265	A-6	276	9.23	30907.89	Cytoplasm. Nucleus.
*AhDREB33*	AHE.Chr09.2064	Chr09:31042796–31,044,004	A-1	246	5.35	26886.1	Cytoplasm. Nucleus.
*AhDREB34*	AHE.Chr09.2065	Chr09:31052890–31,054,293	A-4	267	5.56	28494.66	Cytoplasm. Nucleus.
*AhDREB35*	AHE.Chr10.2047	Chr10:36505720–36,506,559	A-6	279	8.65	30929.66	Nucleus.
*AhDREB36*	AHE.Chr14.596	Chr14:6307226–6,308,452	A-6	408	4.95	45969.67	Nucleus.
*AhDREB37*	AHE.Chr15.1123	Chr15:14600920–14,603,232	A-6	397	9	44356.83	Cytoplasm. Nucleus.
*AhDREB38*	AHE.Chr16.1115	Chr16:13775047–13,776,768	A-6	396	8.77	44153.72	Nucleus.
*AhDREB39*	AHE.Chr16.1118	Chr16:13812025–13,813,747	A-6	396	8.77	44153.72	Nucleus.
*AhDREB40*	AHE.Chr17.1004	Chr17:28120730–28,121,799	A-4	270	5.55	29544.82	Nucleus.
*AhDREB41*	AHE.Chr17.1180	Chr17:29983075–29,984,043	A-6	322	9.02	35424.62	Nucleus.
*AhDREB42*	AHE.Chr17.1423	Chr17:32597833–32,598,768	A-1	226	5.17	25160.04	Cytoplasm. Nucleus.
*AhDREB43*	AHE.Chr17.1426	Chr17:32612394–32,613,411	A-1	207	5.6	23143.08	Cytoplasm. Nucleus.
*AhDREB44*	AHE.Chr17.1617	Chr17:35015250–35,016,186	A-1	226	5.17	25160.04	Cytoplasm. Nucleus.
*AhDREB45*	AHE.Chr17.1619	Chr17:35039968–35,040,728	A-4	182	4.93	19104.44	Nucleus.
*AhDREB46*	AHE.Chr17.286	Chr17:14797803–14,799,624	A-2	321	6.14	35757.96	Nucleus.
*AhDREB47*	AHE.Chr18.165	Chr18:2089806–2,090,504	A-1	211	5.13	23363.08	Nucleus.
*AhDREB48*	AHE.Chr18.166	Chr18:2093861–2,094,762	A-1	235	5.27	25906.96	Cytoplasm. Nucleus.
*AhDREB49*	AHE.Chr18.169	Chr18:2134958–2,135,593	A-1	211	4.85	23291.2	Cytoplasm. Nucleus.
*AhDREB50*	AHE.Chr18.170	Chr18:2141155–2,141,715	A-1	186	10.87	21898.34	Nucleus.
*AhDREB51*	AHE.Chr18.179	Chr18:2217284–2,218,030	A-1	201	5.57	22376.97	Cytoplasm. Nucleus.
*AhDREB52*	AHE.Chr18.187	Chr18:2341530–2,342,069	A-4	179	4.99	19121.48	Nucleus.
*AhDREB53*	AHE.Chr18.189	Chr18:2381623–2,382,162	A-4	179	4.87	19002.29	Nucleus.
*AhDREB54*	AHE.Chr18.465	Chr18:5350537–5,352,190	A-6	326	8.87	35786.22	Nucleus.
*AhDREB55*	AHE.Chr18.491	Chr18:5614621–5,616,274	A-6	326	8.87	35786.22	Nucleus.
*AhDREB56*	AHE.Chr18.766	Chr18:9221856–9,222,987	A-4	273	5.5	29747.02	Cytoplasm. Nucleus.
*AhDREB57*	AHE.Chr19.1202	Chr19:17676186–17,676,818	A-6	210	9.56	23387.32	Nucleus.
*AhDREB58*	AHE.Chr19.2	Chr19:38198–38,596	A-5	132	9.71	14913.18	Chloroplast.Cytoplasm.
*AhDREB59*	AHE.Chr20.1355	Chr20:23665663–23,667,517	A-6	251	9.01	27979.5	Nucleus.
*AhDREB60*	AHE.Chr20.4	Chr20:57654–59,105	A-5	187	10.51	21904.94	Cytoplasm.
*AhDREB61*	AHE.Chr20.692	Chr20:8007217–8,007,846	A-4	209	9.95	22430.44	Nucleus.
*AhDREB62*	AHE.Chr21.2113	Chr21:32491889–32,493,910	A-6	345	5.49	38322.07	Nucleus.
*AhDREB63*	AHE.Chr21.793	Chr21:7728009–7,729,232	A-6	407	4.76	45316.1	Cytoplasm. Nucleus.
*AhDREB64*	AHE.Chr22.1042	Chr22:11903441–11,906,057	A-6	432	7.68	48632.16	Nucleus.
*AhDREB65*	AHE.Chr22.1627	Chr22:20415877–20,417,805	A-2	197	9.46	20709.22	Nucleus.
*AhDREB66*	AHE.Chr22.665	Chr22:6829285–6,830,752	A-6	407	4.76	45301.08	Nucleus.
*AhDREB67*	AHE.Chr23.1106	Chr23:11110494–11,111,202	A-6	197	8.34	22145.94	Nucleus.
*AhDREB68*	AHE.Chr23.674	Chr23:6445261–6,446,481	A-4	269	4.96	28636.44	Cytoplasm. Nucleus.
*AhDREB69*	AHE.Chr23.861	Chr23:8364788–8,366,681	A-6	219	6.24	24435.47	Nucleus.
*AhDREB70*	AHE.Chr24.1023	Chr24:10798380–10,799,591	A-6	198	8.4	21838.56	Nucleus.
*AhDREB71*	AHE.Chr24.754	Chr24:8114979–8,116,243	A-4	270	4.8	28926.97	Cytoplasm. Nucleus.
*AhDREB72*	AHE.Chr24.774	Chr24:8243565–8,244,830	A-4	270	4.8	28939.02	Cytoplasm. Nucleus.
*AhDREB73*	AHE.Chr25.1287	Chr25:26504762–26,505,963	A-2	304	5.88	33851.88	Nucleus.
*AhDREB74*	AHE.Chr25.56	Chr25:673351–674,202	A-6	283	8.53	32037.42	Nucleus.
*AhDREB75*	AHE.Chr26.378	Chr26:4387488–4,388,120	A-4	210	5.09	21875.09	Cytoplasm. Nucleus.
*AhDREB76*	AHE.Chr26.382	Chr26:4425221–4,425,988	A-4	255	7.63	28273.54	Cytoplasm. Nucleus.
*AhDREB77*	AHE.Chr26.42	Chr26:511918–512,799	A-6	293	6.46	32795.23	Nucleus.
*AhDREB78*	AHE.Chr26.916	Chr26:12098244–12,099,140	A-2	298	6.01	33318.15	Nucleus.
*AhDREB79*	AHE.Chr27.186	Chr27:1895680–1,896,662	A-4	265	5.04	28343.4	Cytoplasm. Nucleus.
*AhDREB80*	AHE.Chr27.187	Chr27:1907201–1,907,908	A-1	235	5.26	25879.08	Nucleus.
*AhDREB81*	AHE.Chr27.702	Chr27:6892622–6,895,142	A-2	367	4.9	40439.86	Nucleus.
*AhDREB82*	AHE.Chr27.736	Chr27:7218622–7,219,716	A-3	364	6.64	39193.96	Nucleus.
*AhDREB83*	AHE.Chr28.794	Chr28:8073594–8,074,703	A-2	369	4.82	40793.23	Nucleus.
*AhDREB84*	AHE.Chr28.843	Chr28:8591686–8,592,783	A-3	365	6.32	39087.85	Cytoplasm. Nucleus.
*AhDREB85*	AHE.fragScaff_scaffold_218_pilon.20	fragScaff_scaffold_218_pilon:270,866–271,708	A-6	280	9.07	30,650	Nucleus.
*AhDREB86*	AHE.original_scaffold_1148_pilon.6	original_scaffold_1148_pilon:26,433–27,662	A-6	409	4.8	45512.31	Cytoplasm. Nucleus.
*AhDREB87*	AHE.original_scaffold_348_pilon.3	original_scaffold_348_pilon:50,437–53,037	A-6	430	6.62	48429.9	Nucleus.
*AhDREB88*	AHE.original_scaffold_611_pilon.21	original_scaffold_611_pilon:325,847–327,469	A-4	251	8.66	27724.86	Cytoplasm. Nucleus.
*AhDREB89*	AHE.original_scaffold_611_pilon.23	original_scaffold_611_pilon:352,854–353,483	A-4	209	5.52	21935.19	Cytoplasm. Nucleus.
*AhDREB90*	AHE.original_scaffold_697_pilon.10	original_scaffold_697_pilon:66,344–66,862	A-5	172	9.14	19004.68	Cytoplasm. Nucleus.
*AhDREB91*	AHE.original_scaffold_708_pilon.26	original_scaffold_708_pilon:243,515–244,063	A-5	182	7.87	20612.87	Cytoplasm. Nucleus.
*AhDREB92*	AHE.original_scaffold_771_pilon.15	original_scaffold_771_pilon:94,336–94,821	A-5	161	5.64	17269.1	Cytoplasm. Nucleus.
*AhDREB93*	AHE.original_scaffold_851_pilon.6	original_scaffold_851_pilon:147,620–148,496	A-6	198	9.48	21740.36	Nucleus.

To explore the phylogenetic relationships between *DREB* genes in jackfruit and other plants, a phylogenetic tree of the *DREB* family was constructed using *A. heterophyllus* Lam. (93 members), *Arabidopsis thaliana* (L.) Heynh (63 members), and *F. vesca* (32 members) (Fig. 3B). The results showed that the *DREB* genes of the three species were divided into five subgroups (I–V), with 27, 13, 19, 22, and 12 AhDREB genes in each subgroup, respectively. Subgroups I and V had the most and least *DREB* genes, respectively. In addition, compared with strawberries, AhWRKY genes were closely related to Arabidopsis WRKY family members, indicating that AhWRKY genes are more closely related to monocotyledonous plants.

### Gene structure and conserved motif analysis of the AhDREB gene family

**Fig. 4:**
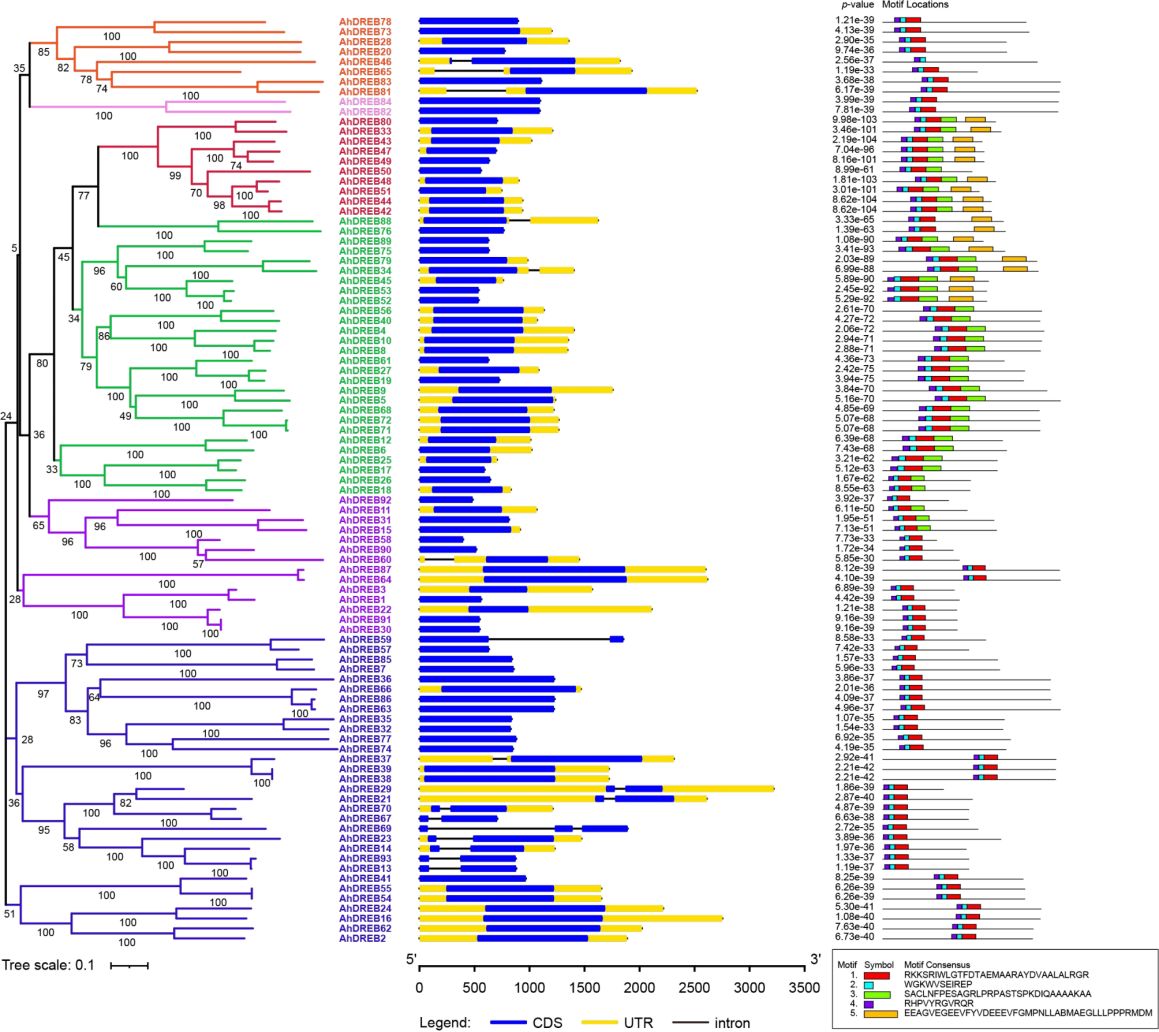
Phylogenetic relationships, gene structure cluster, conserved motif analysis of the *AhDREB* family The AhDREB protein are classified into six subfamily. Different subfamilies are represented by different colors. Different conserved motifs indicate blocks of different colors and sizes

The exon/intron distribution was analyzed to better understand the AhDREB function and its structural characteristics. All but eleven *AhDREB* genes contained at least one exon; of the exceptions, *AhDREBs* 13, 14, 21, 23, 29, 46, 59, 67, 70, and 93 had two exons, and one *AhDREB* (69) had three exons (Fig. 4).

In addition, five conserved motifs of AhDREB proteins were predicted through the MEME website and named Motifs 1–5 (Fig. 4). Motifs 1, 2, and 4 were detected in all AhDREB proteins except AhDREB46, which lacked Motif 1. The results indicated that Motifs 1, 2, and 4 were related to the *AP2* family conserved domain.

### Chromosomal localization and synteny analysis of the AhDREB gene family

**Fig. 5:**
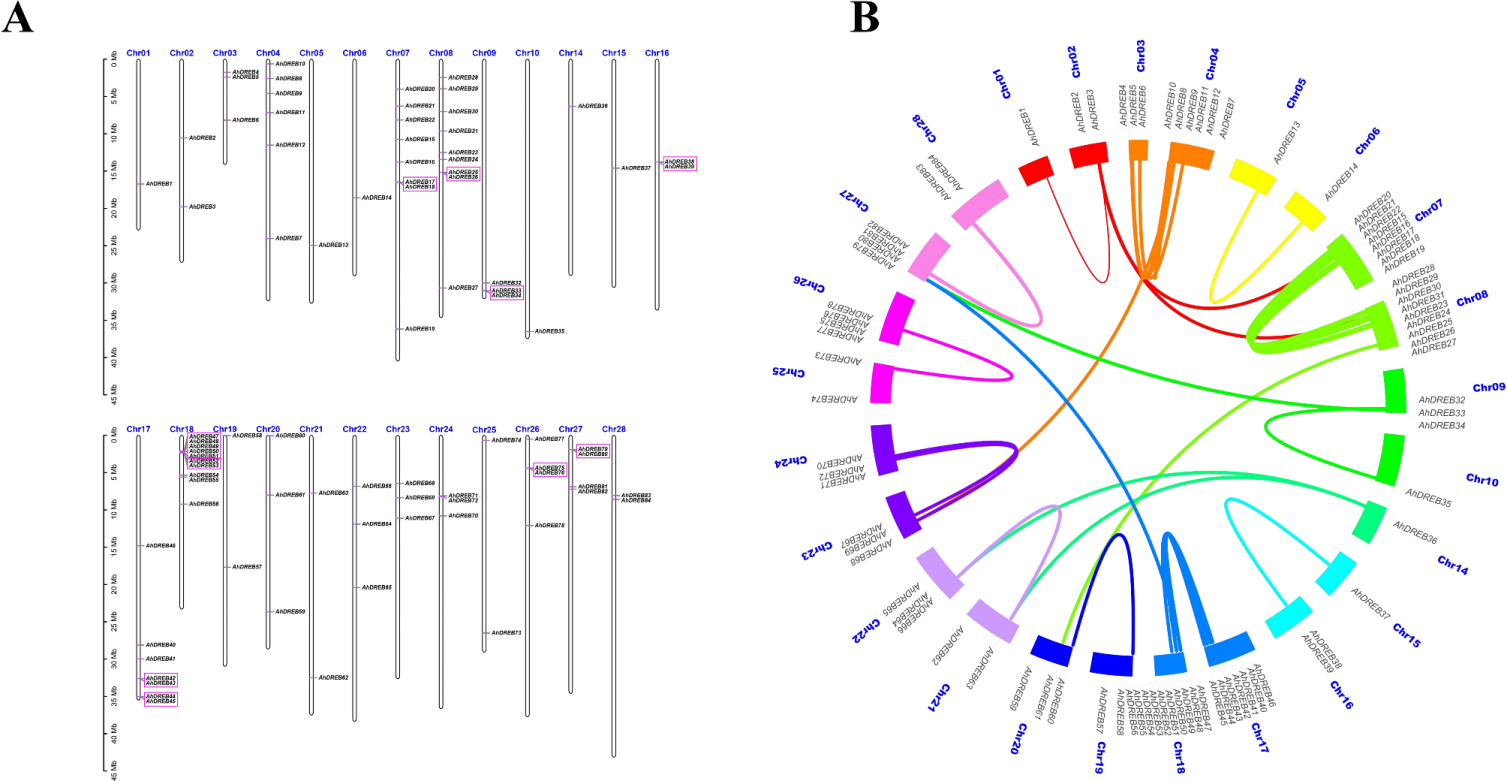
Chromosomal distribution and gene duplication event collinearity of the *DREB*family. (**A**) Chromosomal locations of the *DREB* family. Pink frames represent tandem duplications. (**B**) Gene duplication event collinearity of the *DREB* family. The Circos map drawn by TBtools shows the position relationship of 42 pairs of tandem duplications detected in jackfruit genome. The different color lines represent collinearity pairs among the *DREB* family inside the circle

According to the information on chromosome gene position, 93 *AhDREB* members were distributed unevenly on 26 chromosomes (Fig. 5A). Chromosome 18 had the largest number (10) of *AhDREB* genes, whereas Chromosomes 1, 5, 6, 10, 14, and 15 had the smallest number (1). In addition, 10 tandem duplication events involving 23 *AhDREB* genes were observed, namely, *AhDREB17* and *AhDREB18*, *AhDREB25* and *AhDREB26*, *AhDREB33* and *AhDREB34*, *AhDREB38* and *AhDREB39*, *AhDREB42* and *AhDREB43*, *AhDREB44* and *AhDREB45*, *AhDREB47*, *AhDREB48*, *AhDREB49*, *AhDREB50* and *AhDREB51*, *AhDREB52* and *AhDREB53*, *AhDREB75* and *AhDREB76*, and *AhDREB79* and *AhDREB80*. Notably, *AhDREB* was in the tandem cluster of Chromosome 18, which contained seven consecutive aligned members (*AhDREB47*, *AhDREB48*, *AhDREB49*, *AhDREB50*, *AhDREB51*, *AhDREB52*, and *AhDREB53*).

In the plant genome, tandem repeat events are the primary factors driving plant evolution and are crucial in species diversity. The One Step MCScanX function of TBtool software was used to analyze gene fragment replication events in AhDREB genes (Fig. 5B). The results revealed 42 tandem duplications involving 84 *AhDREB* genes, which were distributed on 25 chromosomes. In addition, the abundant segmental duplication events indicated that the AhDREB family genes were possibly generated by segmentation or tandem duplication, which may have favored the evolution and expansion of the *AhDREB* family.

### Expression profile analyses of the AhDREB gene family in jackfruit leaves under cold stress

**Fig. 6:**
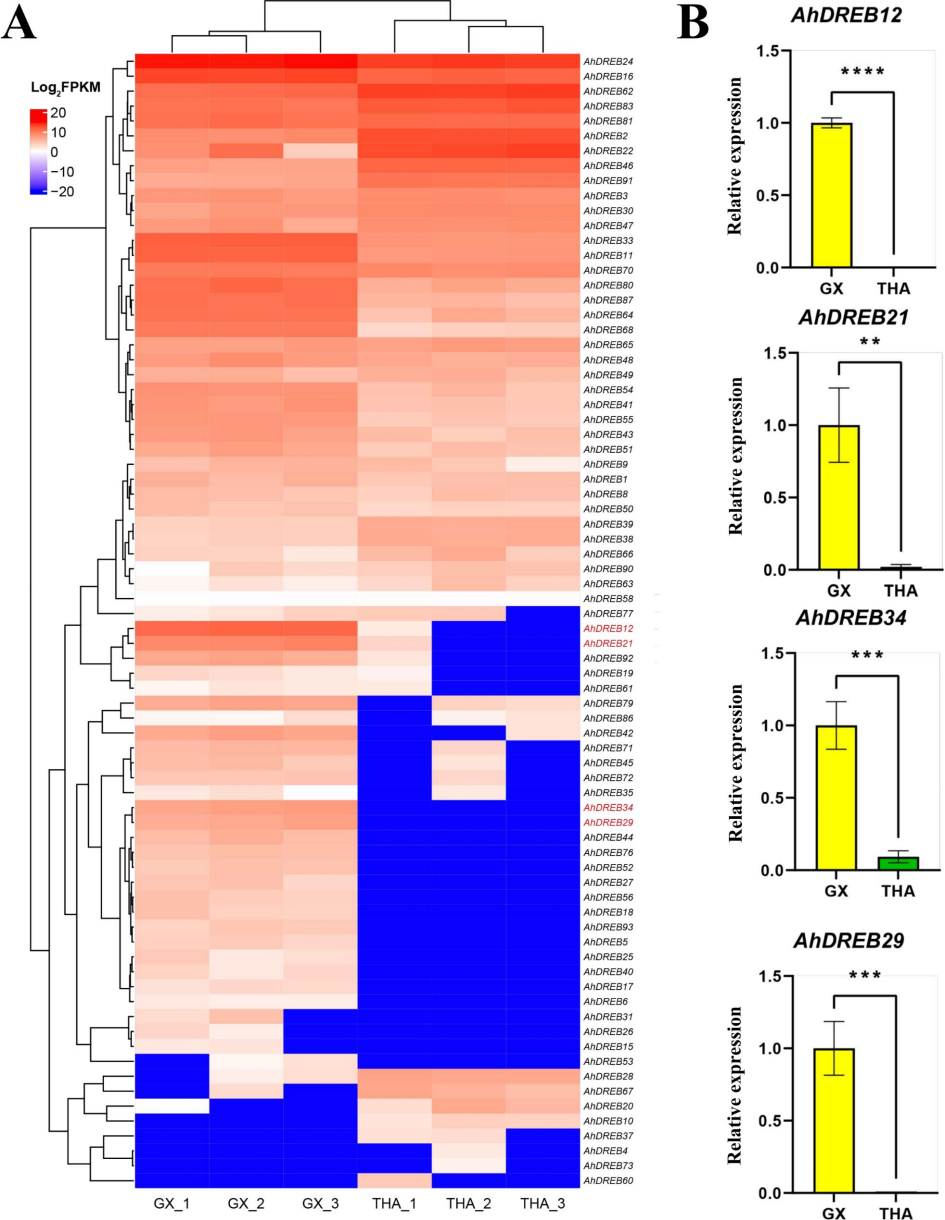
Expression profile of the *DREB* family in ‘GX’ and ‘THA’ under cold stress. (**A**) The heatmap of the *DREB* family and only four biological replication was used for ‘GX’ and ‘THA’. (**B**) The expression profiles of *AhDREB12*, *AhDREB21*, *AhDREB29*, and *AhDREB34* in ‘GX’ and ‘THA’ under cold stress, as revealed by qRT − PCR. The data were calculated by the 2−∆∆Ct method. The internal reference gene was Tubulin, and the error line represented the standard deviation (*n* = 3). (**p < 0.01, ***p < 0.001, and ****p < 0.0001)

A study has shown that parts of the *DERB* family are affected by various abiotic stress responses in plants [34]. RNA sequence datasets from ‘GX’ and ‘THA’ plants exposed to cold stress conditions were used to investigate the expression profiles of the *AhDREB* family. Most *AhDREB* genes had higher expression levels in ‘GX’ leaves than they did in ‘THA’ leaves (69%), suggesting the significance of the *AhDREB* family in cold stress. Notably, *AhDREB12*, *AhDREB21*, *AhDREB29*, and *AhDREB34* were differentially expressed in the ‘GX’ and ‘THA’ leaves under cold stress (Fig. 6). The results indicated that the resistance to cold stress of the four *AhDREB* genes in ‘GX’ might be stronger than that in ‘THA.’

For a detailed analysis of the resistance of the four *AhDREB* genes to cold stress in ‘GX’ and ‘THA,’ qRT − PCR was used to measure the expression of *AhDREB12*, *AhDREB21*, *AhDREB29*, and *AhDREB34* in ‘GX’ and ‘THA’ exposed to cold stress. The expression levels of *AhDREB12*, *AhDREB21*, *AhDREB29*, and *AhDREB34* were significantly down-regulated in ‘THA,’ and the four *AhDREB* genes, almost without expression, consistent with the RNA-seq dataset. Moreover, these results further confirmed that the resistance to cold stress of the four *AhDREB* genes in the ‘GX’ is stronger than that in ‘THA.’

## Discussion

### Physiological differences between ‘GX’ and ‘THA’ under cold stress

Unpredicted variability in temperature is associated with sudden cold stress events, and cold stress is a significant factor that limits the development, distribution, and production of plants and even leads to plant death [7, 35]. Photosynthetic capacity is closely related to plant yield and quality; however, the photosynthetic process is sensitive to temperature stress. Therefore, it is used to assess the ability of plants to cope with stress [36]. In this study, CF imaging was used to analyze the photosynthetic state of jackfruit varieties ‘GX’ and ‘THA,’ and the results showed that Fvʹ/Fmʹ, Fqʹ/Fmʹ, and NPQ in GX were markedly stronger than those in THA. Cold stress affects the absorption, conversion, and electron transfer of light energy, resulting in excess excitation energy accumulation in the reaction center of Photosystem II and damage to the photosynthetic mechanism, reducing Fvʹ/Fmʹ, Fqʹ/Fmʹ, and NPQ and leading to the decline of photosynthetic capacity [37, 38]. Hence, we concluded that ‘GX’ adapts to cold stress more than ‘THA’ does.

When plants are subjected to cold stress, a series of signaling, gene regulation, metabolic pathways, and other reactions are triggered to regulate their growth, development, and metabolic status to adapt to cold environments [39]. Under natural selection, different populations of the same plant have different genetic resources. In this study, ‘GX’ and ‘THA’ had differences in cold resistance and gene expression levels, and 30,585 DEGs were identified. Transcriptome functional enrichment showed that the DEGs were enriched in various metabolism-related pathways, including “tyrosine metabolism,” “starch and sucrose metabolisms,” “phenylpropanoid biosynthesis,” “phenylalanine metabolism,” “metabolic pathways,” “carbon metabolism,” and “biosynthesis of secondary metabolites.” A study has shown that the cold tolerance of *Erianthus fulvus* was related to starch and sucrose metabolic pathways [40]. In the comparison of cold-tolerant and cold-sensitive varieties of *Brassica napus*, many differential metabolites and genes were related to starch and sucrose metabolisms [41]. This suggests that the difference in cold tolerance between ‘GX’ and ‘THA’ may be related to the accumulation of some carbohydrates. Starch and sucrose are carbohydrates, and carbohydrates are the primary products of photosynthesis. The DEGs of ‘GX’ and ‘THA’ were significantly enriched in “photosynthesis,” “photosynthesis-antenna proteins,” and “carbon metabolism” pathways, further indicating that ‘GX’ and ‘THA’ had different responses to cold stress. In addition, “plant hormone signal transduction” and “mitogen-activated protein kinase (MAPK)” signaling pathway were differentially enriched in ‘GX’ and ‘THA.’ A study has shown that plant perception of environmental conditions triggers stress-specific signal transduction, which can induce whole genome transcriptional reprogramming, inducing other transcriptional mechanisms [42]. Therefore, the difference in cold resistance between ‘GX’ and ‘THA’ may differ from their signal transduction ability.

### AhDREB family is evolutionarily conserved

DREB TFs are members of the AP2/ERF family and are crucial in cold acclimation to attain the maximum freezing tolerance in plants [43]. Increasing evidence suggests that the function of *DREB* genes in cold resistance is conserved in higher plants, and they can specifically bind to CRT/DRE (CCGAC) motifs in cold response gene promoters to activate their expression [10]. In our study, 93 nonredundant *DREB* genes were identified in the jackfruit S10 cultivar. Abundant *DREB* genes have been identified in various plant species; the number of *DREB* genes detected in jackfruit was lower than that in *Triticum aestivum* (204) [44] and higher than that in soybean (*Glycine max*) (73) [45], apple (*Malus pumila* Mill.) (60) [46], and mung bean (*Vigna radiata*) (71) [47]. Gene structure analysis showed that 81.73% of *AhDREB* genes were intron-less, which was lower than those in strawberries (*Fragaria × ananassa* Duch.) [48] and higher than those in rice (*Oryza sativa* L.) [49]. Conserved motif analysis revealed that Motifs 1, 2, and 4 were detected in all AhDREB proteins except AhDREB46, indicating that they might comprise AP2 conserved motifs. Moreover, the same subgroup of *AhDREB* genes in the phylogenetic tree had similar gene structures and conserved motifs, indicating that the same subgroup of *AhDREB* genes possibly had similar functions. The diversification of plants and the expansion of gene families are believed to be caused by whole-genome and tandem duplications [50, 51]. Chromosomal localization and synteny analyses indicated that *AhDREB* genes were unevenly distributed in 25 of the 28 jackfruit chromosomes and in the entire jackfruit genome, and two tandem duplication gene pairs were in the *AhDREB* family. The number of *AhDREB* genes in jackfruit was greater than that in most other plants, possibly owing to the large number of tandem and whole-genome duplication events.

### DREB family genes may be critical regulatory factors of cold stress resistance in jackfruit

TFs are groups of proteins that specifically bind to the promoter *cis*-elements of downstream genes, regulate stress target genes, and ensure the transcription and expression of target genes at a certain time and place, playing a crucial role in plant growth and development and environmental responses [52]. Over 50 TFs, including *AP2/ERF*, *bZIP*, *NAC*, *C2H2*, *MADS*, *MYB*, and *WRKY*, have been identified in plants [53]. A study has shown that the *AP2/ERF* family participates in abiotic stress [54]. In strawberries (*Fragaria × ananassa* Duch.), *FvDREB1* expression was correlated with the early and middle stages of drought stress. However, *FvDREB2* expression was correlated with the middle and late stages of drought stress, indicating that gene expressions under the same conditions differ and that *FvDREB18* regulates the expression of downstream genes in response to drought stress by controlling *FvDREB1* and *FvDREB2* [44]. Qian et al. [55] used transcriptomic data and qRT − PCR analyses to reveal that *Erianthus fulvus* has five and two *DREB* genes that respond to cold and drought stress conditions, respectively. In oil palms (*Elaeis species*), *EgDREB1* participates in signaling communication from root to shoot under mild drought, and ectopic expression of *EgDREB1* in transgenic tomatoes enhances the expression of reactive oxygen species (ROS)-related genes under PolyethyleneGlycol (PEG) treatment and cold stress [56]. Sheng et al. showed that alfalfa (*Medicago sativa* L.) *DREB* genes, which were cold-stress responsive, were significantly downregulated in the cold-resistant alfalfa variety. Moreover, eight *DREB* genes were implicated in a long-term freezing-stress adaptation with a great potential [57]. *AnDREB5.1* overexpression improved tolerance to cold, osmotic, and oxidative stress conditions in *Ammopiptanthus nanus* (*A. nanus*). Under cold stress, *AnDREB5.1* overexpression increased antioxidant enzyme activity and inhibited excessive elevation of ROS levels in *Ammopiptanthus nanus* [58]. A recent study showed that *MdDREB2A* can directly bind to the promoter of *MdSWEET12* to promote sucrose transport under drought conditions [59]. *PeDREB28* can directly bind to the promoter of the ABA receptor protein pyrabactin-resistance-like (*DlaPYL3*) gene and activate its expression, suggesting that *PeDREB28* may change the stress resistance of bamboo by regulating the ABA signaling pathway [60]. In our study, most *AhDREB* genes were up-regulated in ‘GX’ under cold stress. DEGs between cold-resistant (‘GX’) and cold-sensitive (‘THA’) varieties were enriched in signal transduction and metabolism pathways. These results suggest that the *DREB*e family may be involved in cold stress response in jackfruit by regulating various signal transduction and metabolic pathways. The results of the study provide significant insights into the response of jackfruit to cold stress, which will reveal the potential molecular mechanism and contribute to the screening and breeding of jackfruit.

## Conclusions

In this study, two jackfruit cultivars (‘GX’ and ‘THA’) were subjected to cold stress. By combining transcriptomics data and chlorophyll fluorescence assay, the DEGs and metabolic pathways involved in response to cold stress were identified. In ‘GX’ and ‘THA,’ phenylalanine metabolism, metabolic pathways, and carbon metabolism were significantly enriched, indicating they might be the basic metabolic pathways in jackfruit for responding to cold stress. DEG analysis showed that the *AP2/ERF* genes had the largest proportion, suggesting the significance of *AP2/ERFs* in increasing cold resistance in jackfruit. Therefore, we analyzed the *DREB*family, the subfamily of *AP2/ERF*family. We identified 93 *DREB* gene family members in the whole genome of *A. heterophyllus*, a detailed investigation of the basic physicochemical properties, phylogenetic, gene structure, conserved motif, synteny, and expression profile of the *AhDREB* gene family in ‘GX’ and ‘THA’ under cold stress was conducted. These findings provide a valuable reference in exploring the crucial gene and breeding of jackfruit cold-resistant varieties.

## Electronic supplementary material

Below is the link to the electronic supplementary material.

**Supplementary Material 1: Supplementary Data 1** Summary of primers used in this study

**Supplementary Material 2: Supplementary Figure 1** Correlation analysis of leaf samples of two jackfruit strains

## Data Availability

The datasets generated and analyzed during the current study are included in this article. The sequencing data that support the findings of this study are openly available in the global pharmacopoeia genome database (http://www.gpgenome.com/species/92). Raw reads for RNA-Seq were downloaded from the NCBI database with accessionnumber SRP129502 (https://www.ncbi.nlm.nih.gov/sra/?term=SRP129502) and SRP092562 (https://www.ncbi.nlm.nih.gov/sra/SRP092562) and the materials used during the current study are available from the corresponding author on reasonable request.

## Abbreviations

BLAST: Basic Local Alignment Search Tool
CBF: C-repeat binding factor
CF: Chlorophyll fluorescence
DEG: Differentially expressed gene
DRE/CRT: Dehydration-responsive element/C-repeat
DREB: Dehydration-responsive element binding protein
GX: Artocarpus heterophyllus Lam. (jackfruit) variety ‘Guangxi’
MEME: Multiple Em for Motif Elicitation
NPQ: Non-photochemical quenching
PCR: Polymerase chain reaction
TF: Transcription factor
THA: Artocarpus heterophyllus Lam. (jackfruit) variety ‘Thailand’
