# Supplementary figures and images for: Transcriptome analysis and genome-wide identification of the dehydration-responsive element binding gene family in jackfruit under cold stress
Source: BMC Genomics. 2024 Sep 4;25:833. doi: 10.1186/s12864-024-10732-1 (PMC11373402; doi:10.1186/s12864-024-10732-1)

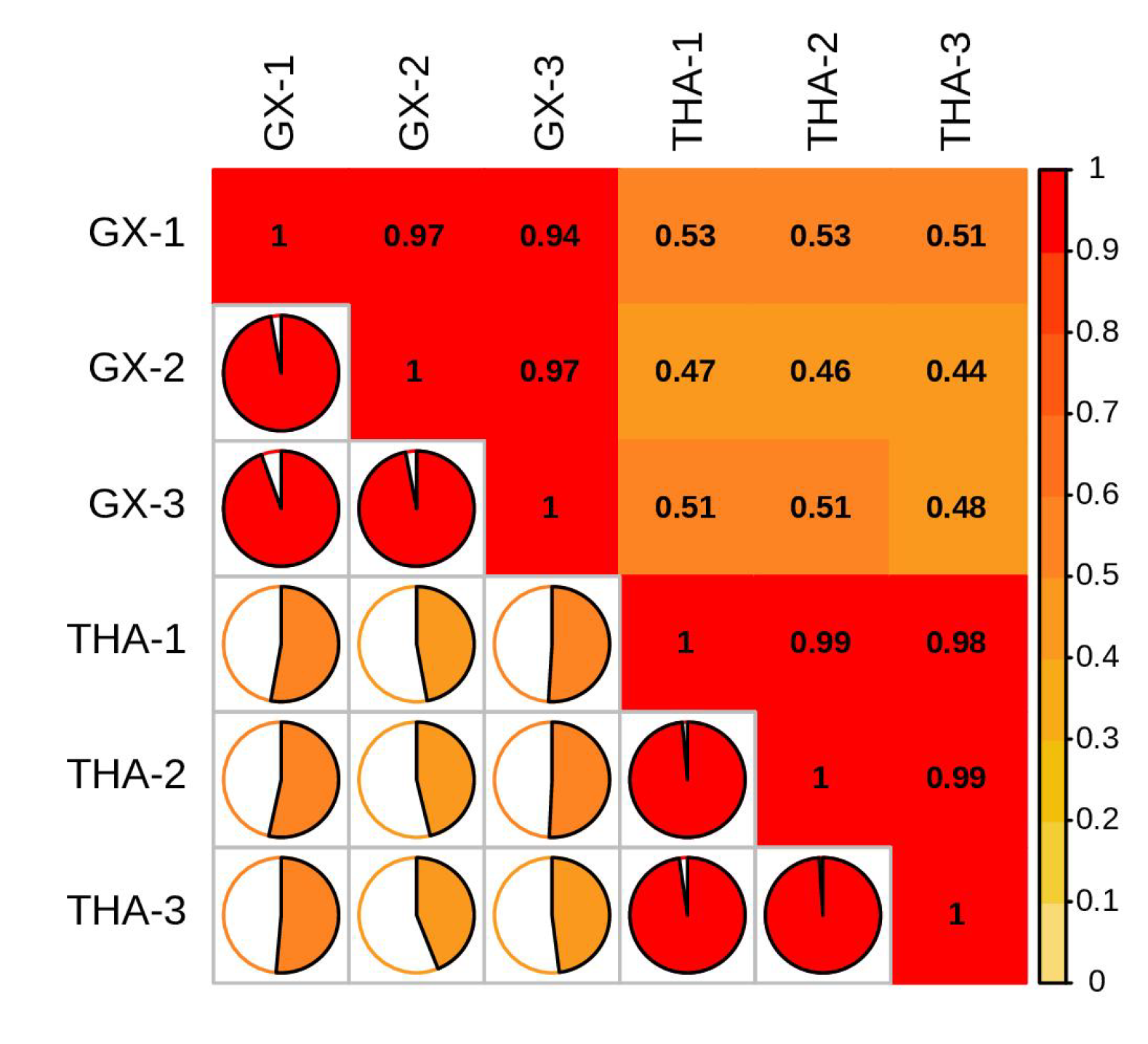

Supplement: Supplementary file 2 — Supplementary Material 2: Supplementary Figure 1 Correlation analysis of leaf samples of two jackfruit strains [file 12864_2024_10732_MOESM2_ESM.png]
